# Electrical, photocatalytic, and sensory properties of graphene oxide and polyimide implanted with low- and medium-energy silver ions

**DOI:** 10.3762/bjnano.16.123

**Published:** 2025-10-13

**Authors:** Josef Novák, Eva Štěpanovská, Petr Malinský, Vlastimil Mazánek, Jan Luxa, Ulrich Kentsch, Zdeněk Sofer

**Affiliations:** 1 Institute of Nuclear Physics of CAS, v.v.i., Husinec - Rez, Rez, 250 68, Czech Republic; 2 Department of Physics, Faculty of Science, University of J. E. Purkyne, Usti nad Labem, 400 96, Czech Republic; 3 Department of Inorganic Chemistry, University of Chemistry and Technology, Prague 6, 166 28, Czech Republic; 4 Ion Beam Center, Helmholtz-Zentrum Dresden Rossendorf, 01328 Dresden, Germany

**Keywords:** ERDA, graphene oxide, ion implantation, photocatalysis, polyimide, RBS

## Abstract

Precise control of electrical conductivity, humidity sensitivity, and photocatalytic activity in polymeric and carbon-based materials is essential for advancing technologies in environmental sensing, flexible electronics, and photocatalytic systems. Conventional chemical modification methods often lack spatial precision, introduce impurities, and risk structural degradation. Ion implantation provides a controllable alternative for tuning surface properties at the nanoscale, enabling the targeted introduction of functional species without chemical reagents. This work investigates the effects of low-energy (20 keV) and medium-energy (1.5 MeV) Ag^+^ ion implantation on the electrical, sensory, and photocatalytic properties of graphene oxide (GO) and polyimide (PI). Implantations were carried out with fluences ranging from 3.75 × 10^12^ cm^−2^ to 1 × 10^16^ cm^−2^. Silver ions offer excellent electrical, catalytic, and plasmonic characteristics, making them ideal for multifunctional enhancement of GO and PI. Elemental and structural changes induced by implantation were analyzed using Rutherford backscattering spectroscopy, elastic recoil detection analysis, Raman spectroscopy, Fourier-transform infrared spectroscopy, and X-ray photoelectron spectroscopy. Surface morphology was assessed via atomic force microscopy. Electrical properties as a function of air humidity were evaluated using a two-point method, and photocatalytic activity was tested by monitoring the UV-induced decomposition of rhodamine B. The results demonstrate that ion implantation significantly reduces surface resistivity and enhances both the photocatalytic activity and humidity sensitivity of GO and PI. The most pronounced improvements occurred at higher fluences, where defect generation and partial deoxygenation contributed to optimal performance. Ion implantation thus represents an effective approach for tuning the multifunctional behavior of polymer systems.

## Introduction

Silver ion implantation is an effective strategy for controlling modification of the physicochemical properties of polymers and graphene-based materials. This method allows for the precise introduction of implanted atoms into the surface layers without the need for subsequent chemical modification, thus opening new possibilities in the development of functional thin films. In this study, Ag ions were used to modify polymer matrices to improve chemical and electronic properties of the modified materials.

In our previous studies, light ions such as Cu [1–2] and C [3] were implanted into GO, PI, and other polymers. In contrast, the implantation of heavier ions like Ag interacts with the target material through different mechanisms. Owing to its higher mass, Ag has a different electronic stopping behavior, which results in a distinct ionization of the surrounding matrix. Furthermore, the chemical reactivity of Ag towards functional groups in polymers differs from that of Cu or C, potentially leading to unique structural modifications and functional responses. Among various metallic ions available for ion implantation, Ag offers a unique combination of properties that make it particularly suitable for simultaneous enhancement of conductivity, humidity sensitivity, and photocatalytic activity [4]. Ag exhibits high electrical conductivity, strong catalytic activity, and pronounced plasmonic effects in the visible spectrum [5]. These synergistic characteristics make Ag ions especially advantageous for multifunctional material modification [6].

Silver is one of the metals with the highest electrical conductivity, and its ion implantation is an effective method for controlled modification of the electrical properties of dielectric and semiconducting materials [7]. When Ag ions are implanted into polymer substrates, such as polyimide (PI) or graphene oxide (GO), fundamental changes occur at the molecular and electronic levels, leading to a significant decrease in the surface electrical resistivity [8].

During implantation, the kinetic energy of the implanted particles is transferred to the matrix, causing local crystallization and carbonization of the polymer structures. This process is associated with the destruction of the original covalent bonds (e.g., C–N, C=O, and C–O) and the simultaneous transformation of the amorphous polymer phase into a graphitized structure with higher electrical parameters [9]. This results in carbon regions rich in conjugated π-electron systems, which allow for efficient charge delocalization and the formation of conducting pathways [10]. The delocalized electrons in these regions can freely pass between neighboring centers, thereby reducing the energy activation for conducting electric current.

At the same time, the aggregation of the implanted Ag ions and the formation of metal islands or clusters in the form of nanostructures can further enhance the electrical conductivity of the non-conductive material [11]. These islands can act as conductive bridges between graphitic regions, and, at sufficient density, form a percolating network that significantly reduces resistance. The resulting hybrid system consisting of a carbonized matrix, conjugated bonds, and silver cluster structures exhibits dramatically higher conductivity than the unmodified material, which can be exploited, for example, for the fabrication of flexible optoelectronics, thin-film electrodes, or electroactive sensors [12].

In addition to the increase in conductivity, the implantation of silver ions can also significantly affect the interaction of the material with water molecules, thereby improving its sensing response to changes in air humidity [13]. By modifying the chemical composition and microstructure of the surface, the affinity of the material for air humidity is altered, which is crucial for the design of thin-film humidity sensors [14].

In the case of both GO and PI, Ag ion implantation can cause partial deoxygenation and redistribution of functional groups (especially C–O, C=O, and N–H), thereby changing the hydrophilic character of the surface [15]. At the same time, however, polar groups, which are crucial for the adsorption of water molecules, are not eliminated; on the contrary, their spatial arrangement and chemical accessibility may be favored by implantation. This results in a higher adsorption capacity and a faster response to changes in relative humidity [16].

In addition to improving the affinity for water vapor, the implantation of Ag ions also translates into changes in optical and photochemical properties of the material, such as photocatalytic properties [17]. The modified electronic structure, characterized by a narrowing of the bandgap and an increased density of states near the Fermi level, promotes absorption in the visible region and facilitates the generation of electron–hole (e^−^–h^+^) pairs upon light irradiation. The silver clusters also exhibit plasmonic resonances, which amplify the local electromagnetic field and promote the generation of excited states with longer lifetimes [18].

Photocatalysis is a surface-driven process in which the absorption of photons with energy equal to or greater than the material’s bandgap results in the generation of e^−^–h^+^ pairs. These photogenerated charge carriers migrate to the surface, where they initiate redox reactions with adsorbed species, such as water or dissolved oxygen, leading to the formation of reactive oxygen species that degrade organic contaminants. In Ag-modified polymer matrices, several synergistic effects contribute to enhanced photocatalytic activity. The catalytically active sites resulting from the carbonization of the polymer matrix, together with the silver clusters formed during ion implantation, provide suitable conditions for reactant adsorption and efficient charge transfer. Silver ions exhibit strong localized surface plasmon resonance in the visible spectral range, enhancing light absorption and enabling the utilization of a broader portion of the solar spectrum [19]. These combined effects result in a significantly higher photocatalytic efficiency of implanted samples compared to their non-implanted samples, both in terms of reaction rate and quantum yield. Such properties are promising for applications including photocatalytic water purification, self-cleaning surfaces, and light-activated antimicrobial coatings [20].

The elemental composition of the modified films was characterized by Rutherford backscattering spectrometry (RBS) and elastic recoil detection analysis (ERDA). The other analytical methods used were Raman spectroscopy, Fourier-transform infrared spectroscopy and X-ray photo-electron spectroscopy (XPS). The electrical properties were investigated by the two-point method. The photocatalytic properties were tested in a dark chamber by disintegration of rhodamine B using UV light. The experimental results clearly confirmed that silver ion implantation led to a significant reduction in the surface electrical resistance of both GO and PI. Simultaneously, an enhancement of photocatalytic activity was observed, demonstrated by a faster degradation of rhodamine B compared to the non-implanted samples.

## Results and Discussion

### Projected range and energy loss simulation by SRIM

During implantation, the ions interact with atomic nuclei or their electron shells, which results in a loss of the ions’ kinetic energy. The energy losses of the implanted ions are due to electronic stopping power (*S*_e_) and nuclear stopping power (*S*_n_) [4]. Electronic stopping most often leads to the excitation of electrons to higher energy levels or to the ionization of the atoms and contributes to the formation of free radicals [21]. In contrast, nuclear stopping most often leads to the displacement of the target nuclei and to significant defect formation in the irradiated material, such as substitution defects [21].

**Figure 1:**
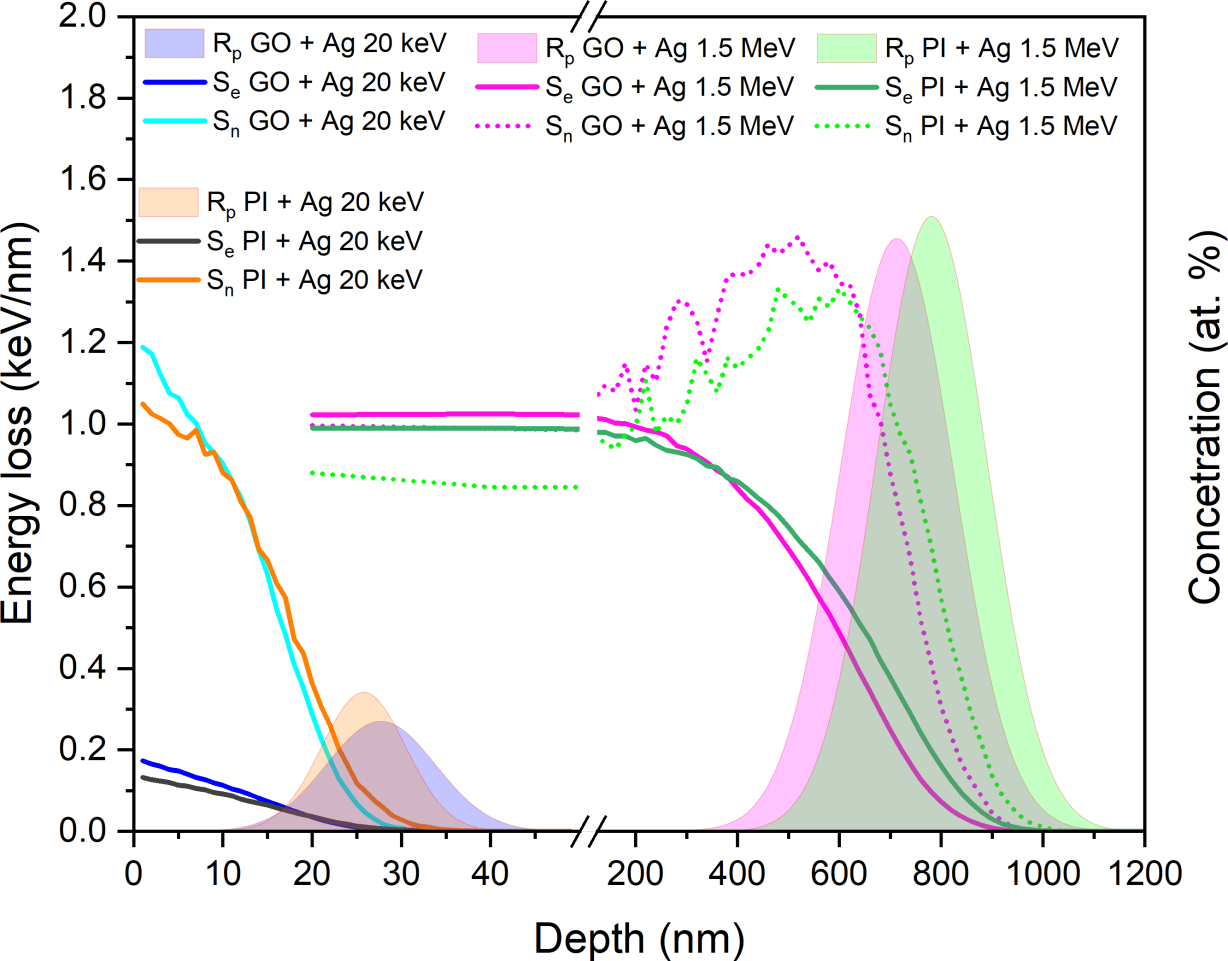
SRIM-calculated depth distribution of ranges of Ag ions with energies of 20 keV and 1.5 MeV in GO and PI and Ag ions’ nuclear stopping powers (*S*_n_) and electronic stopping powers (*S*_e_) in the same matrix.

We performed an initial estimation of the ions’ energy losses and range depth of the implanted Ag ions in GO and PI films using SRIM software [22]. This calculation was performed through a complex Monte Carlo simulation. The initial GO composition for SRIM simulation was determined by RBS analysis of the pristine sample, while the composition and density of the PI polymer were obtained from the SRIM database. The results of the SRIM simulation are shown in Table 1 and Figure 1. Table 1 presents the mean projected range *R*_p_ and straggling of projected range of the Ag ions in GO and PI, together with the nuclear and electron stopping powers and their ratio at the sample surface. It is evident from Table 1 that, for low-energy implantation, the projected ranges *R*_p_ of Ag ions are 27.4 and 25.6 nm for GO and PI, respectively. The projected range of Ag ions with energy of 1.5 MeV is up to 26 times higher (for both GO and PI) compared to ion implantation with an energy of 20 keV.

**Table 1:** Ratios of nuclear and electronic stopping powers of Ag ions with energies of 20 keV and 1.5 MeV in GO and PI and their projected ranges (*R*_p_) in GO and PI.

	*S*_n_/*S*_e_	*R*_p_ (nm)	Δ*R*_p_ (nm)

GO – 20 keV	8.16	27.4	5.8
PI – 20 keV	8.96	25.6	5.8
GO – 1.5 MeV	3.59	706.8	100.1
PI – 1.5 MeV	3.34	764.2	106.1

At 20 keV, the *S*_n_/*S*_e_ ratio is high (approximately 8–9), indicating that nuclear collisions dominate the energy loss mechanism. This results in intense atomic displacements near the surface, leading to significant surface damage, defect formation, and localized modifications within a shallow depth (*R*_p_ = 25–27 nm). The opposite situation occurs when both GO and PI were implanted with 1.5 MeV Ag ions. In this case, the electron stopping powers dominate near the surface. At 1.5 MeV, the *S*_n_/*S*_e_ ratio is lower (3.3–3.6), meaning that electronic interactions dominate, especially in the initial stages of penetration. The ions penetrate much deeper (*R*_p_ = 700–760 nm). At this energy, the implantation primarily affects subsurface layers, leading to bulk modifications with more gradual structural changes and less surface sputtering. The details of the energy stopping powers at the depth of ion implantation are given in Figure 1.

### Elemental characterization by RBS and ERDA

**Figure 2:**
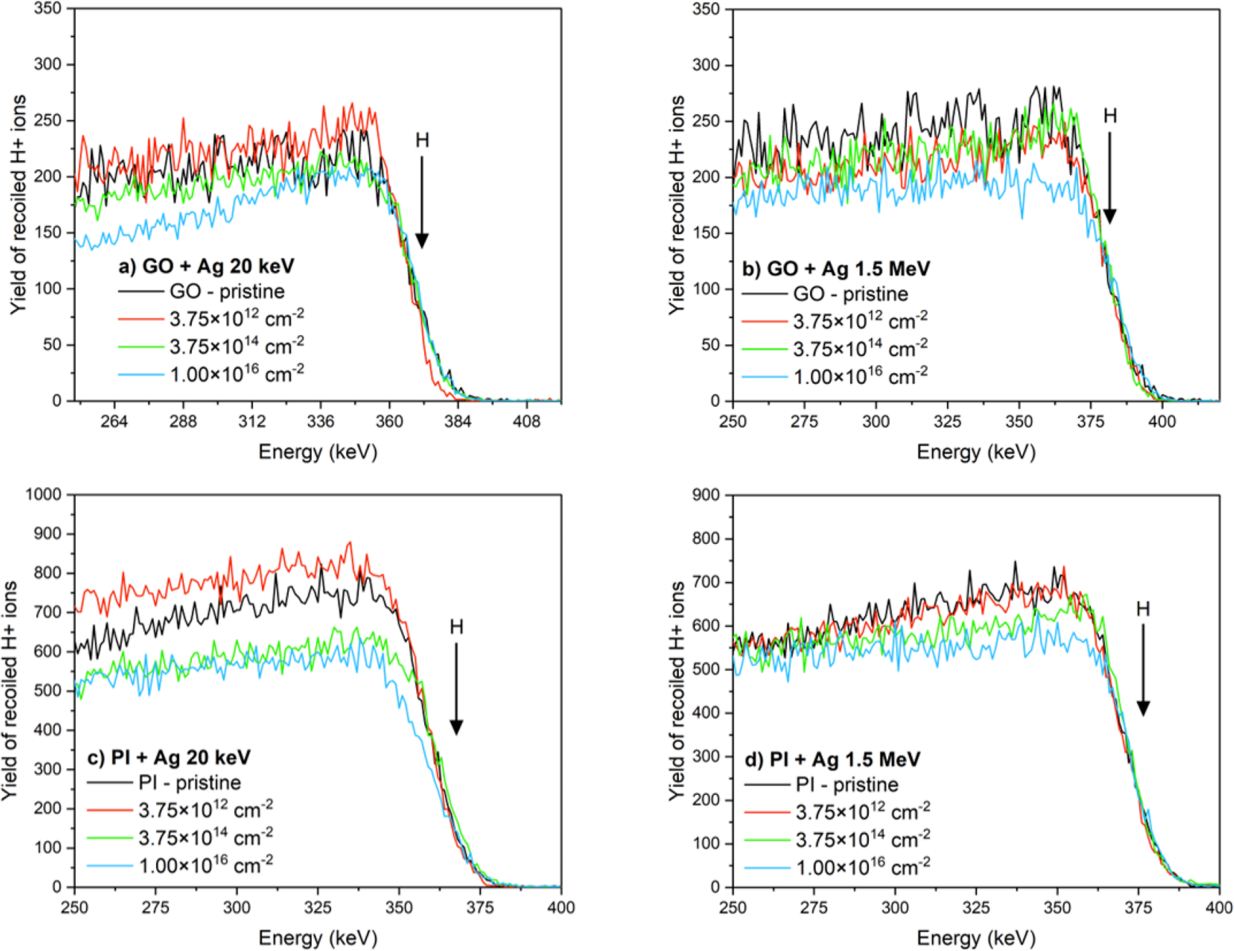
The ERDA spectra of pristine and implanted samples. (a) GO 20 keV Ag ions, (b) GO 1.5 MeV Ag ions, (c) PI 20 keV Ag ions, and (d) PI 1.5 MeV Ag ions with ion fluences of 3.75 × 10^12^ cm^−2^, 3.75 × 10^14^ cm^−2^ and 1 × 10^16^ cm^−2^.

**Figure 3:**
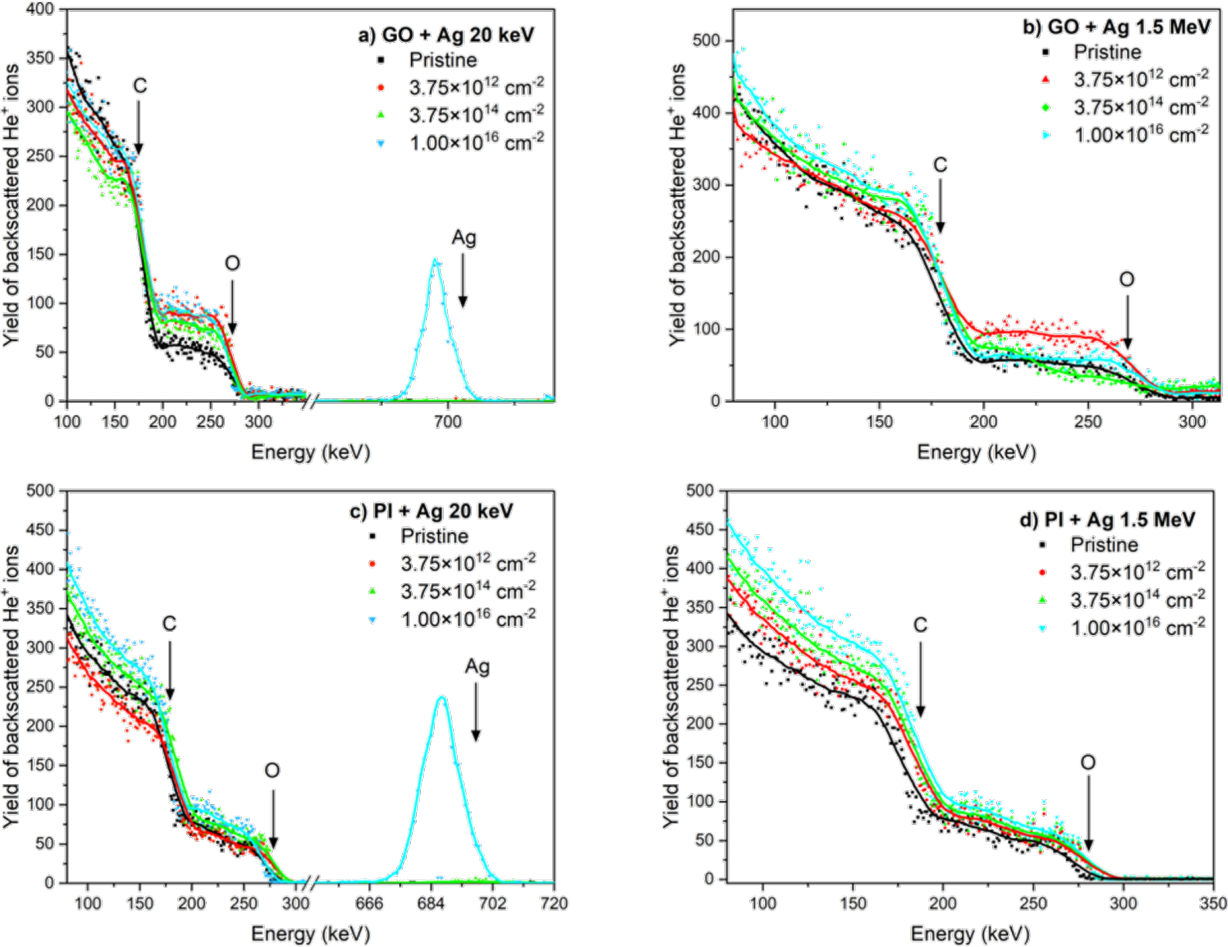
RBS spectra of pristine and implanted samples. (a) GO 20 keV Ag ions, (b) GO 1.5 MeV Ag ions, (c) PI 20 keV Ag ions, and (d) PI 1.5 MeV Ag ions with ion fluences of 3.75 × 10^12^ cm*^−^*^2^, 3.75 × 10^14^ cm*^−^*^2^ and 1 × 10^16^ cm*^−^*^2^.

The elemental composition of GO and PI foils, pristine as well as implanted with Ag ions with low (20 keV) and medium (1.5 MeV) energies, was analyzed by RBS and ERDA spectroscopic methods and the resulting spectra can be seen in the Figure 2. The details are summarized in Table 2. One can see that the 20 keV Ag ion implantation with an ion fluence of 3.75 × 10^12^ cm^−2^ causes in GO the C/O ratio to decrease from 6.2 (for pristine GO) to 3.6 (Table 2 and Figure 3a). There is also an evident increase in the atomic concentration of oxygen for all used ion fluences. For example, in the case of an ion fluence of 3.75 × 10^14^ cm^−2^, it increases by 7 atom %. More pronounced changes in atomic concentration are evident for GO implanted with Ag ions with an energy of 1.5 MeV. There is an obvious gradient where the atomic concentration of carbon increases slightly with increasing ion fluence to 71 atom % for an ion fluence of 1 × 10^16^ cm^−2^. This increase is accompanied by a slight decrease in the atomic concentration of oxygen and hydrogen, which decreases by 3 atom %, as indicated in Table 2 and Figure 3b.

**Table 2:** Elemental composition of GO and PI before and after Ag ion implantation determined using RBS and ERDA.

	20 keV				1.5 MeV			
	C [atom %]	O [atom %]	H [atom %]	C/O	C [atom %]	O [atom %]	H [atom %]	C/O

GO – pristine	68 ± 1.6	11 ± 0.3	20 ± 1.0	6.2	68 ± 1.6	11 ± 0.3	21 ± 1.0	6.2
GO – 3.75 × 10^12^ cm^−2^	65 ± 1.5	18 ± 0.5	20 ± 1.0	3.6	68 ± 1.6	12 ± 0.3	20 ± 1.0	5.6
GO – 3.75 × 10^14^ cm^−2^	68 ± 1.6	17 ± 0.5	15 ± 0.7	3.6	70 ± 1.6	10 ± 0.3	20 ± 1.0	7.0
GO – 1 × 10^16^ cm^−2^	69 ± 1.6	18 ± 0.5	13 ± 0.6	3.8	71 ± 1.7	12 ± 0.3	18 ± 0.9	5.9

PI – pristine	55 ± 1.3	19 ± 0.5	26 ± 1.3	2.9	55 ± 1.3	19 ± 0.5	26 ± 1.3	2.9
PI – 3.75 × 10^12^ cm^−2^	52 ± 1.2	20 ±0.5	28 ± 1.4	2.6	54 ± 1.3	21 ± 0.6	25 ± 1.2	2.6
PI – 3.75 × 10^14^ cm^−2^	58 ± 1.4	21 ± 0.6	21 ± 1.0	2.7	58 ± 1.4	21 ± 0.6	21 ± 1.0	2.8
PI – 1 × 10^16^ cm^−2^	59 ± 1.4	22 ± 0.6	19 ± 0.9	2.7	64 ± 1.5	22 ± 0.6	14 ± 0.7	2.9

Next, we studied PI implanted with 20 keV Ag ions. It is clear from Figure 3c and Table 3 that there are only negligible changes in the atomic oxygen concentration for all ion fluences. There is a slight increase in the atomic concentration of carbon (by 4 atom % for ion fluence 1 × 10^16^ cm*^−^*^2^). More significant changes were achieved in the case of ion implantation of PI by 1.5 MeV Ag ions (Figure 3d), where there was an increase in the atomic concentration of carbon for an ion fluence 3.75 × 10^14^ cm*^−^*^2^ to a value of 58 atom %. An even more significant increase is seen for ion fluence 1 × 10^16^ cm*^−^*^2^, where the atomic concentration of carbon reached 64 atom %, which is about 9 atom % more than in pristine PI.

**Figure 4:**
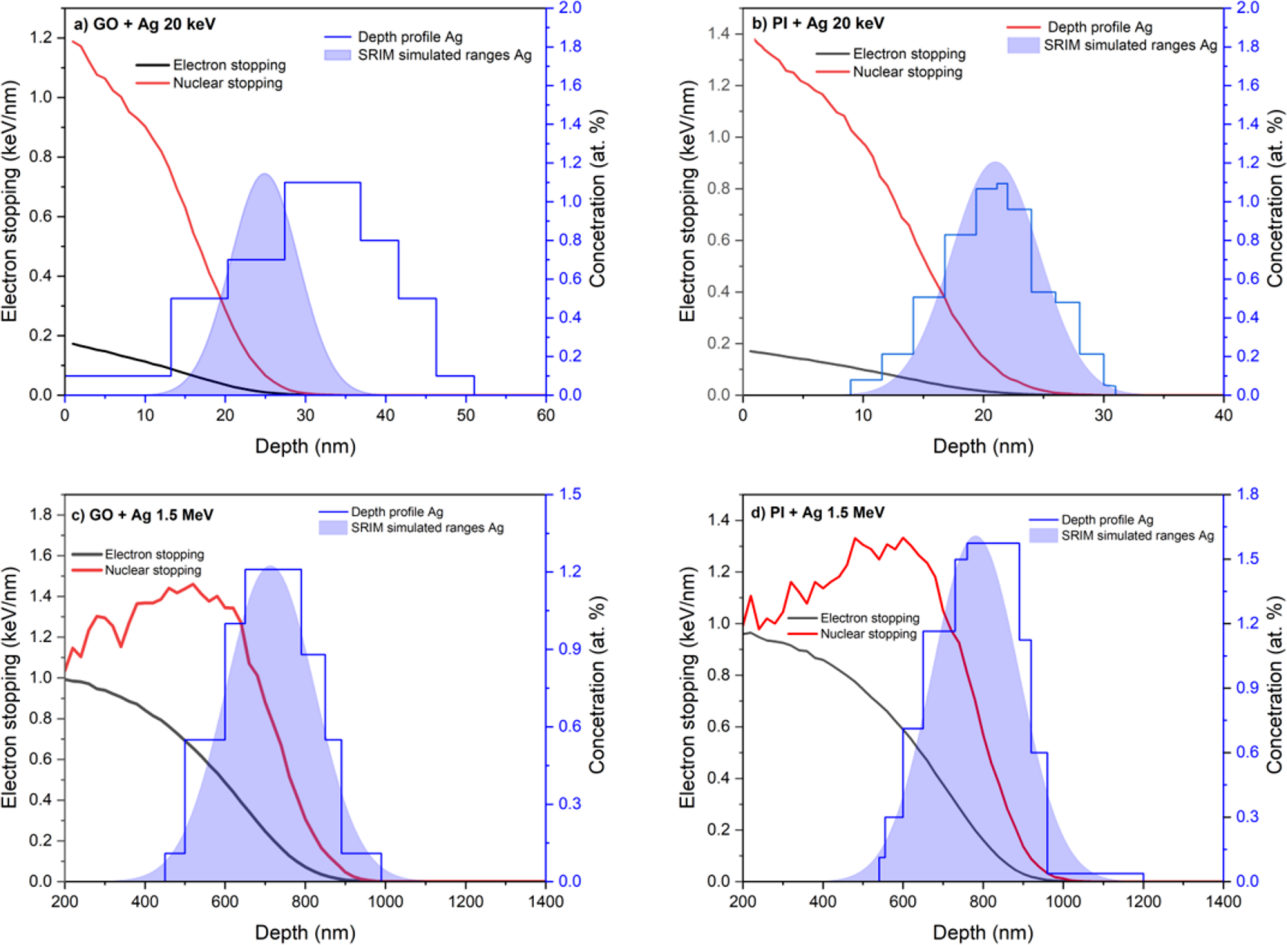
Simulated electron and nuclear stopping from SRIM with simulated Ag ion range compared with RBS derived depth profiles for (a) GO 20 keV, (b) PI 20 keV, (c) GO 1.5 MeV, and (d) PI 1.5 MeV.

The simulated Ag depth profiles obtained through SRIM were compared with the Ag concentration depth profiles determined via RBS using 2.0 MeV He ions, as illustrated in Figure 4 for GO and for PI implanted with 20 keV and 1.5 MeV Ag ions for an ion fluence of 1 × 10^16^ cm*^−^*^2^. The SRIM-calculated projected ranges of Ag ions with an energy of 1.5 MeV show reasonable agreement with the Ag concentration depth profiles in Figure 4a for GO and Figure 4b for PI. At this depth of ion penetration, we anticipate the most significant disruption of the carbon structures in both materials, involving the formation of vacancies and Ag ion interstitials. The discrepancy between the SRIM-simulated and RBS-measured Ag depth profiles can be attributed to several factors. SRIM simulations are based on an idealized model that assumes a homogeneous, amorphous target with constant density and does not account for structural or chemical changes occurring during ion implantation. In reality, Ag^+^ implantation into GO and PI induces carbonization, changes in local density, and possible diffusion or agglomeration of Ag atoms, which can lead to a redistribution of silver compared to the initial ballistic projection. Moreover, surface sputtering and the increase in surface roughness during implantation may result in a shallower experimental profile than predicted. Differences can also arise from the finite depth resolution of RBS, which is influenced by energy straggling and multiple scattering effects, leading to a broadening of the detected profile.

### Surface chemical analysis by XPS

**Figure 5:**
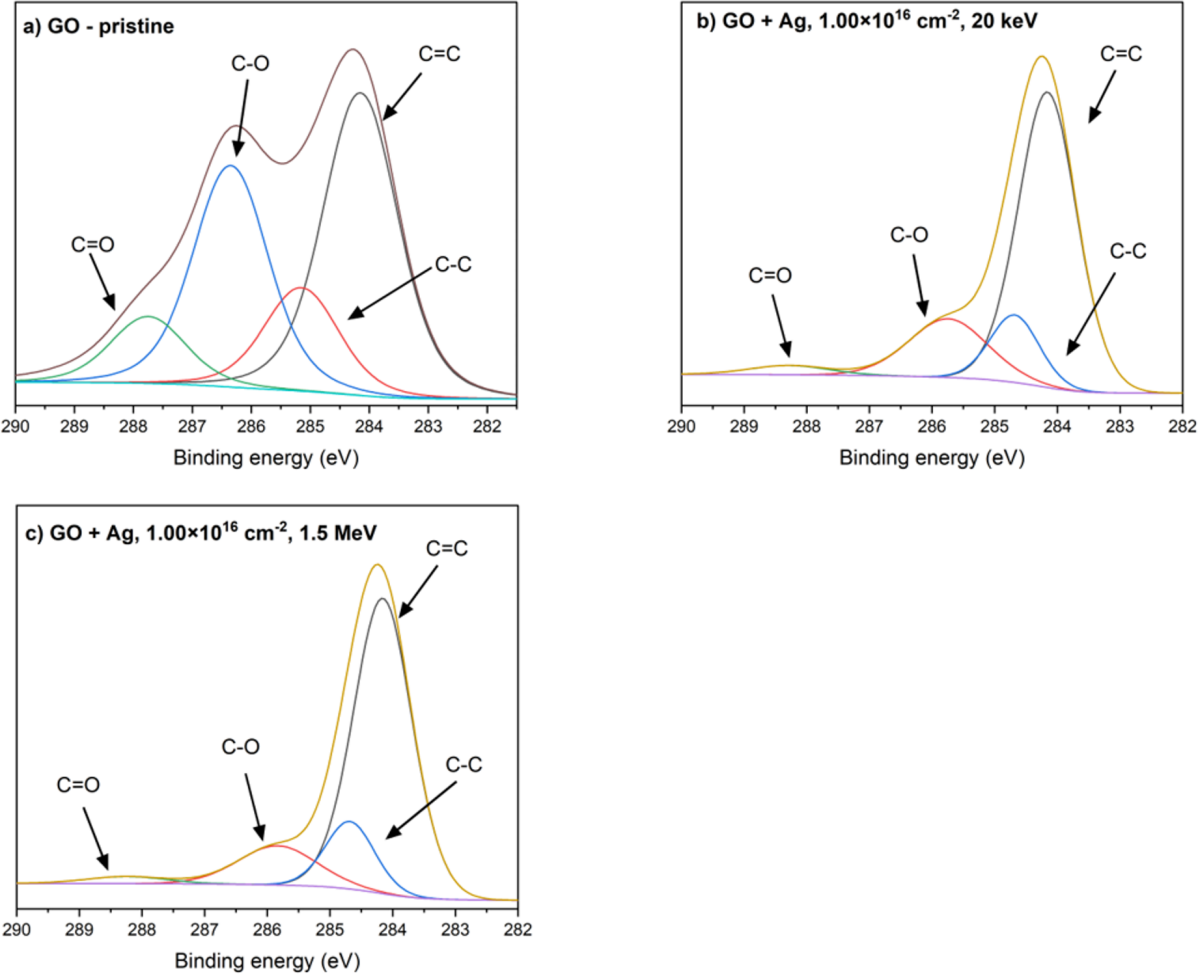
Deconvolution of the XPS C 1s peak of (a) pristine GO, (b) GO implanted with 20 keV Ag ions with an fluence of 1 × 10^16^ cm^−2^, and (c) GO implanted with 1.5 MeV Ag ions with a fluence of 1 × 10^16^ cm^−2^.

**Figure 6:**
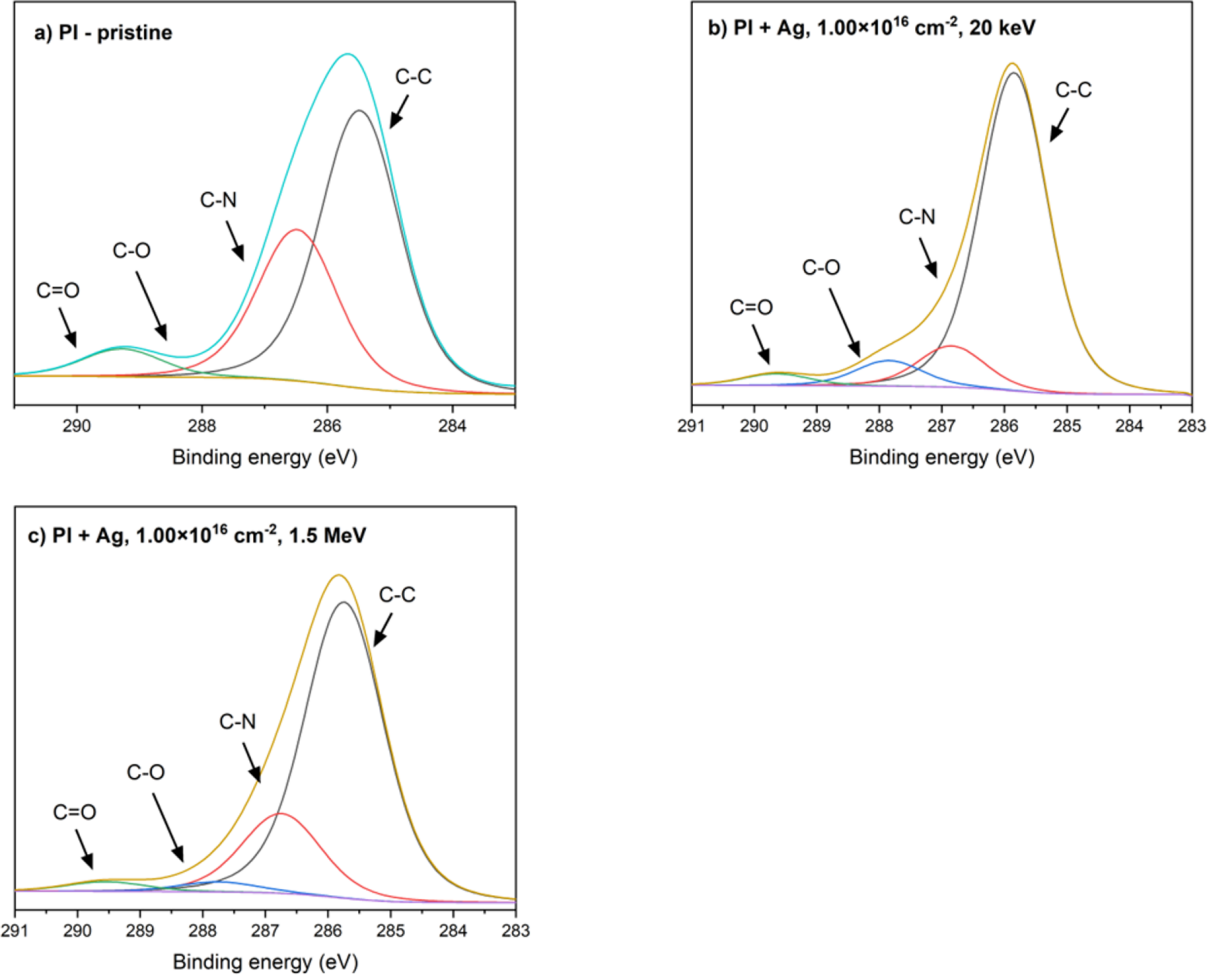
Deconvolution of the XPS C 1s peak of (a) pristine PI, (b) PI implanted with 20 keV Ag ions with a fluence of 1 × 10^16^ cm^−2^, and (c) PI implanted with 1.5 MeV Ag ions with a fluence of 1 × 10^16^ cm^−2^.

Chemical groups and the concentration of chemical elements on the surfaces of GO and PI before and after 20 keV and 1.5 MeV Ag ion implantation were analyzed by X-ray photoelectron spectroscopy (XPS). The XPS method specifically provides information from a thin surface layer with a depth of a few nanometers. The deconvolution of the high-resolution C 1s peak of XPS spectra is illustrated in Figure 5 for GO and in Figure 6 for PI. Spectra are shown for unmodified samples and for samples exposed to the highest ion fluence (1 × 10^16^ cm*^−^*^2^). More detailed information describing the presence of each functional group is given in Table 3.

**Table 3:** The fractions of the individual components of the C 1s peak in pristine and irradiated GO and PI foils with 20 keV and 1.5 MeV Ag ions.

GO	C–C	C=C	C–O	C=O

GO – pristine	42.99	14.68	31.42	9.42
GO – 1 × 10^16^ cm*^−^*^2^ – 20 keV	66.65	17.73	12.78	2.83
GO – 1 × 10^16^ cm*^−^*^2^ – 1.5 MeV	71.31	12.69	13.68	2.31

PI	C–C	C–N	C–O	C=O
PI – pristine	64.12	24.84	2.94	8.11
PI – 1 × 10^16^ cm*^−^*^2^ – 20 keV	71.37	14.40	8.79	5.44
PI – 1 × 10^16^ cm*^−^*^2^ – 1.5 MeV	74.87	20.17	2.54	2.42

The deconvolution of C 1s peak of XPS spectra of GO pristine (Figure 5a) sample shows a fairly balanced proportion of different chemical bonds. The dominant bonds are C–C (42.99%) and C–O (31.42%), which correspond to the structure of graphene oxide where carbon atoms form the basic lattice, while oxygen is present in the form of epoxy and hydroxy groups [23]. The C=O bonds (9.42%) represent carbonyl groups, while C=C bonds (14.68%) indicate the presence of residual conjugated sp^2^-hybridized regions, which are characteristic of oxidized graphene. This structure is the result of an oxidation process that leads to the addition of oxygen functional groups to the surface of graphene and, at the same time, a partial disruption of its original sp^2^-hybridized structure. The relatively high proportion of C–O and C=O bonds indicate a significant oxidation of the surface [24–25].

After implantation of GO with Ag ions at an energy of 20 keV (Figure 5b), significant changes in chemical composition occur. The proportion of C–C bonds increased significantly to 66.65%, while C–O bonds decreased to 12.78% and C=O bonds to 2.83%. This change indicates a reduction in oxygen functional groups, which is due to radiation damage induced by ion implantation. The high ion fluence caused the destruction of carbon–oxygen bonds, leading to partial deoxidation of the surface and an increase in the proportion of carbon networks. At the same time, the proportion of C=C bonds increased to 17.73%, indicating a partial recovery of conjugated sp^2^-hybridized regions, probably due to the rearrangement of carbon atoms after the destruction of oxygen groups. This process is consistent with increasing carbonization and the formation of a carbon network with higher electron density.

Further changes in the chemical structure of GO occur when silver ions with higher energy of 1.5 MeV are implanted (Figure 5c). The proportion of C–C bonds increased to 71.31%, reflecting the ongoing carbonization process and removal of oxygen functional groups. The C–O (13.68%) and C=O (2.31%) bonds remain at low levels, indicating almost complete deoxidation of the surface. At the same time, the proportion of C=C bonds decreased to 12.69%. The reason might be a higher degree of damage to the original sp^2^-hybridized regions due to the higher implantation energy.

The deconvolution of the C 1s region in high-resolution X-ray photoelectron spectroscopy (XPS) spectra of pristine PI is depicted in Figure 6a, illustrating four distinct carbon components. These components include C–C carbons originating from the aromatic rings of the oxydianiline portion of PI observed at 285.86 eV, C–N carbons corresponding to carbon atoms covalently bonded to nitrogen within the imide and amide functionalities of PI, identified at 286.86 eV, C–O carbons bonded to an ether group (287.86 eV), and C=O carbons bonded within the imide ring (289.66 eV) [26–27].

After implantation with 20 keV silver ions, the proportion of C–C bonds increased significantly to 71.37%, indicating the cleavage or rearrangement of oxygen groups, which decreased to 5.44%. At the same time, the proportion of C–N bonds decreased to 14.40%, which may be due to the loss of some secondary bonds or changes in the molecular structure. These changes indicate the onset of radiation degradation of the material, in which oxygen functional groups are removed and the polymer structure rearranges towards a more carbonaceous network.

After implantation with higher-energy ions of 1.5 MeV, the changes were even more pronounced. The proportion of C–C bonds increased to 74.87%, while C–O and C=O bonds decreased slightly to 2.42% and 2.54%, respectively. This decrease confirms that higher implantation energy causes more intense degradation of oxygen functional groups and rearrangement of chemical structure. This results in the formation of a material with a high density of C–C bonds, indicating the formation of a structure that is close to carbonaceous networks. These changes suggest that the implantation of silver ions causes radiation damage, which results in the cleavage of oxygen groups and the formation of free radicals and their subsequent recombination [26].

The results obtained from the XPS analysis, in conjunction with the RBS findings, reveal a consistent trend indicative of ongoing carbonization processes occurring in the surface layers of the PI samples. The reduction in oxygen-containing bonds, such as C–O and C=O, as evidenced by XPS, aligns with the decrease in oxygen concentration detected by RBS, particularly at higher fluences and implantation energies. This deoxidation of the PI surface is a direct consequence of the ion implantation process, where oxygen atoms are likely displaced or removed through radiation-induced breaking of chemical bonds. The increase in C–C bonds observed via XPS distinctly highlights structural rearrangements within the polymer. This suggests the breaking of aromatic benzene rings present in the PI structure, which results in the formation of a more interconnected carbon network. Such changes have significant implications for the material’s properties, particularly its electrical conductivity, as a denser carbon structure typically lowers resistivity and enhances conductive pathway.

### Structure analysis by Raman and FTIR spectroscopies

Comprehensive structural analysis of GO before and after ion irradiation was conducted using Raman spectroscopy [28]. This spectroscopic technique, known for its effectiveness in probing disorders and defects within crystal structures, proved particularly valuable in characterizing graphite and its derivatives [29]. The Raman spectra of graphene oxide usually show three distinct peaks, a D peak, which lies at a wavenumber of 1344 cm^−1^, a G peak at a wavenumber of 1591 cm^−1^, and a 2D band which beginning at a wavenumber of 2356 cm^−1^ [6].

**Figure 7:**
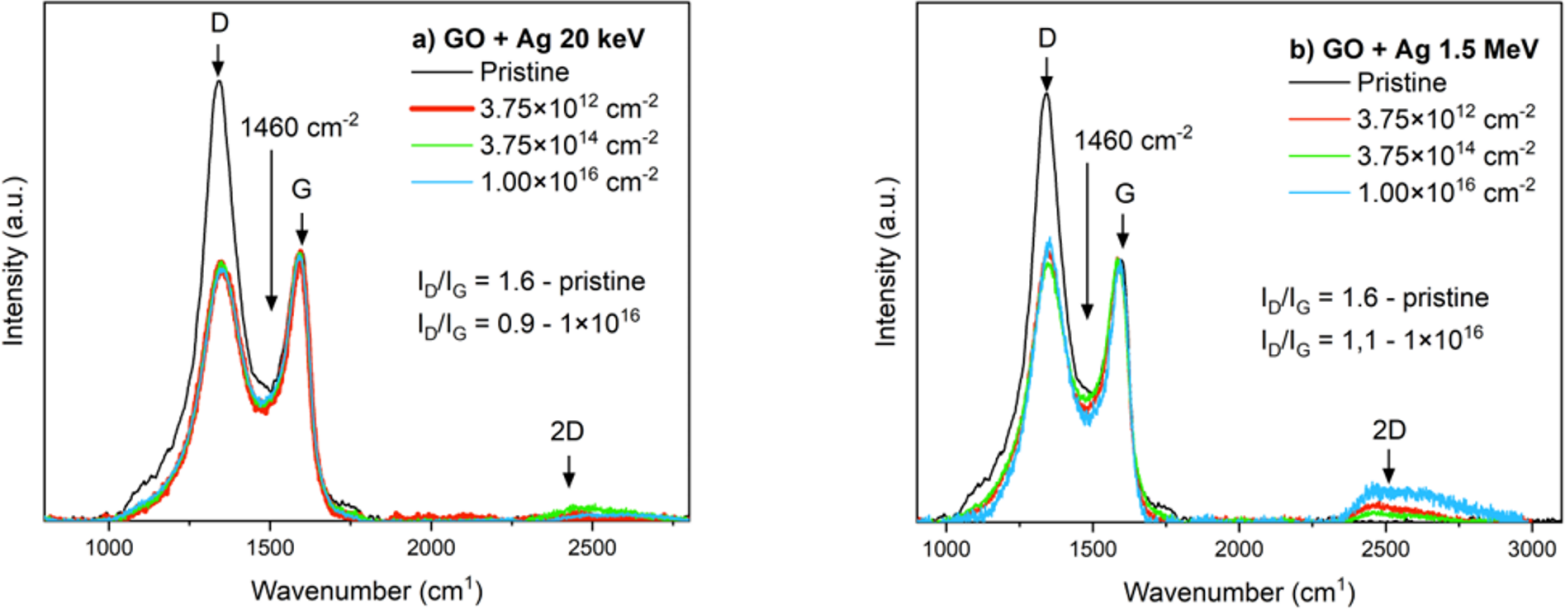
The Raman spectra of pristine and irradiated samples. (a) GO 20 keV and (b) 1.5 MeV Ag ions.

The Raman spectrum in Figure 7 has been normalized to the G peak. According to Figure 7a, irradiation of GO with 20 keV Ag ions leads to significant changes in the structure and vibrational states of GO. One can see a significant decrease in the D peak; this decrease is the same for arbitrary ion fluences, and the *I*_D_/*I*_G_ ratio reaches values of 0.94. The D peak in the Raman spectrum of GO characterizes the degree of defects and structural disturbances in the carbon lattice.

Similar results were obtained when GO was irradiated with 1.5 MeV Ag ions, with a significant decrease in the D peak. In this case, the decrease is most pronounced for the 3.75 × 10^14^ cm^−2^ ion fluence, with the *I*_D_/*I*_G_ ratio reaching values of 1.06, which is higher than in the previous case. Ag ion irradiation causes more defects in GO when 1.5 MeV energy ions were used. By comparing of the Figure 7a and Figure 7b, we can see that both ion fluences lead to a narrowing of the D and G bands with respect to unmodified GO. This narrowing can be induced by structural changes occurring in GO due to ion irradiation. Cracking and breakage of the internal structure of GO may be the most common cause of this profile narrowing [4].

The significant decrease in the intensity of the D peak at both implantation energies, normalised to the G peak, indicates a decrease in defect density in the GO carbon lattice and a reduction in oxygen functional groups. The XPS results confirm this, as there was a significant reduction in oxygen bonds (C–O and C=O) at the highest ion fluence, with C–C bonds becoming the dominant bonds. This trend is also consistent with the RBS measurements. The RBS results showed that, at higher energies of 1.5 MeV, there is a greater removal of oxygen from the surface layer, confirming the deoxidation of GO. This process is reflected in the Raman spectrum as a narrowing of the D and G peaks, suggesting that the implantation causes a rearrangement of the carbon atoms into a structure with lower defect density.

**Figure 8:**
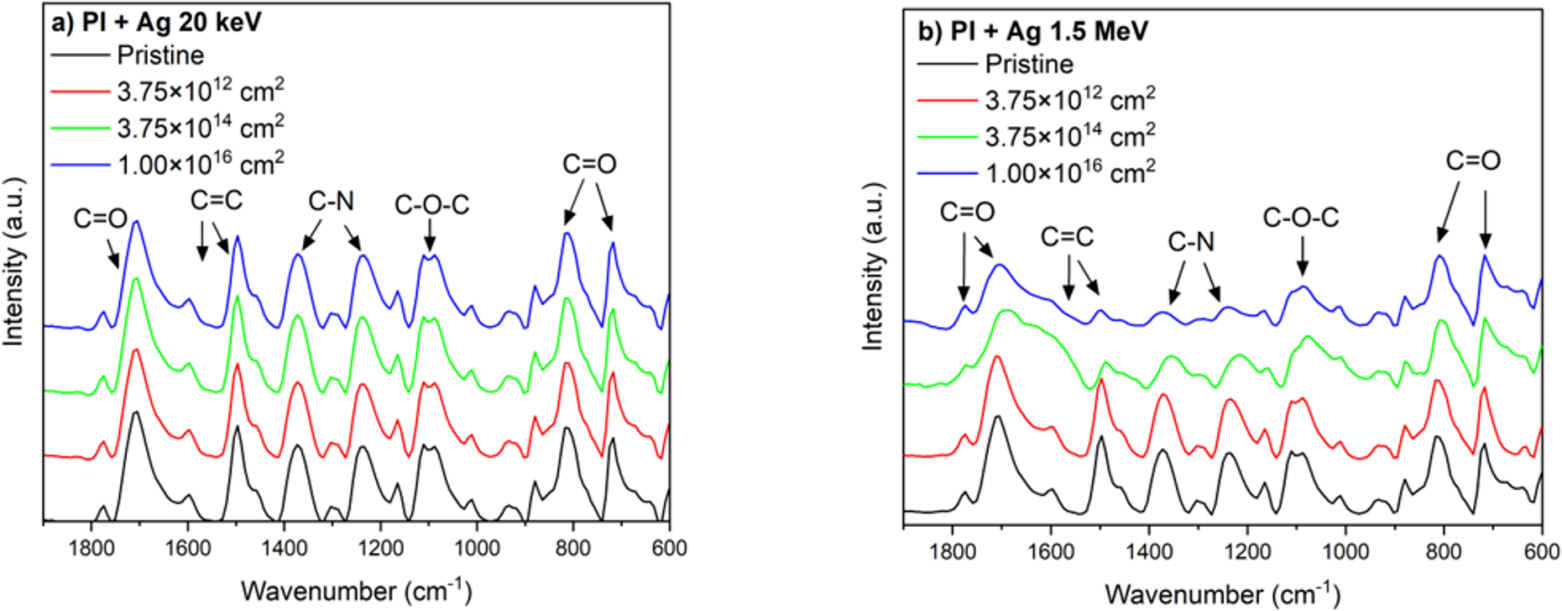
FTIR analysis of PI implanted with Ag ions with ion fluences of 3.75 × 10^12^ cm^−2^, 3.75 × 10^14^ cm^−2^, and 1 × 10^16^ cm^−2^ with energies of (a) 20 keV and (b) 1.5 MeV.

Since the Raman spectra of PI polymers exhibit a high fluorescence background due to strong optical absorption and excitation of π-electrons in phenyl rings, the structural changes in PI were analyzed by Fourier-transform infrared spectroscopy (FTIR) [30] (Figure 8). The FTIR spectra were normalized to the signal at 715 cm^−1^. The spectra consist of several regions of interest, namely, the 715 cm^−1^ wavenumber region corresponding to C=O bonding and the 1083 cm^−1^ peak attributed to C–O–C asymmetrical stretching. Aromatic C–N bonds associated with the imide linkage are located at a wavenumber of 1350 cm^−1^. The wavenumber of 1500 cm^−1^ is attributed to C=C aromatic stretching, indicating the presence of aromatic rings in the polymer backbone. The penultimate significant wavenumber is 1706 cm^−1^, describing C=O symmetrical stretching; finally, the C=O asymmetrical stretching lies at a wavenumber of 1774 cm^−1^, representing the carbonyl group in the imide structure [31–32].

In PI implanted with 20 keV Ag ions (Figure 8a) at low ion fluence, the polyimide structure undergoes minimal changes. At an ion fluence of 3.75 × 10^14^ cm^−2^, there is a slight increase in C=O bonds, indicating a restructuring of the carbonyl groups. However, this trend changes with a fluence of 1 × 10^16^ cm^−2^; there is a slight decrease that indicates initial degradation. Furthermore, with increasing ionic fluence, there is a very slight decrease in C–N and C–O–C vibrations.

Different changes can be observed in the case of implantation of 1.5 MeV Ag ions. At the lowest fluence of 3.75 × 10^12^ cm^−2^, an increase in intensity was observed for the C=O (≈1800 cm^−1^) and C=C (≈1650 cm^−1^) vibrations. This increase indicates a strengthening of these chemical bonds, probably due to the restructuring and formation of new carbonyl and aromatic bonds as a result of ion implantation. At the same time, slight decreases in intensity were observed for the vibrations of CH_2_ (≈1400 cm^−1^), C–N (≈1200 cm^−1^), and C–O–C (≈1100 cm^−1^), indicating the initial cleavage of aliphatic, amide, and ether bonds.

More pronounced changes occur at an average fluence of 3.75 × 10^14^ cm^−2^. The vibrational intensity of C=O (≈1800 cm^−1^) and C=C (≈1650 cm^−1^) decreases significantly, indicating the destruction of carbonyl and aromatic structures. This degradation is due to the higher energy transferred by the ions, leading to fragmentation of the molecules and formation of defects. The C–N (≈1200 cm^−1^) vibrations show a further decrease in intensity, indicating cleavage of aliphatic chains and disintegration of amide groups. The C–O–C (≈1100 cm^−1^) and C=O (≈800 cm^−1^) regions show a significant decrease in signal, indicating more extensive cleavage of ether and carbonyl bonds. These changes can be explained by a combination of physical bond cleavage and chemical changes such as defect formation and local carbonization.

The highest fluence of 1 × 10^16^ cm^2^ causes major changes in the spectrum. The vibrations of C=O (≈1800 cm^−1^) and C=C (≈1650 cm^−1^) are significantly reduced, corresponding to a significant destruction of carbonyl groups and aromatic structures. Similarly, the C–N (≈1200 cm^−1^) signal is minimal, indicating extensive degradation of aliphatic and amide groups. The C–O–C (≈1100 cm^−1^) and C=O (≈800 cm^−1^) regions show lower intensity compared to irradiation with the lower fluences, indicating partial loss of ether and carbonyl bonds. These changes are due to the high dose of energy transferred by the ions, leading to a significant amorphization of the material and probably to the carbonization of the polyimide.

### Surface morphology studied by AFM

Changes in the surface morphology of PI implanted with 20 keV and 1.5 MeV Ag ions at different fluences were examined by atomic force microscopy (AFM). The basic parameters arithmetic average height (*R*_a_) and mean roughness (RMS) are listed in Table 4 [25].

**Table 4:** AFM results describing the basic parameters RMS and *R*_a_ of GO and PI samples before and after ion implantation.

	20 keV		1.5 MeV	

	RMS [nm]	*R*_a_ [nm]	RMS [nm]	*R*_a_ [nm]

GO – pristine	154	119	154	119
GO – 3.75 × 10^12^ cm^−2^	102	95	102	73
GO – 3.75 × 10^14^ cm^−2^	381	235	121	96
GO – 1 × 10^16^ cm^−2^	590	254	69	60
PI – pristine	125	97	125	97
PI – 3.75 × 10^12^ cm^−2^	64	45	69	53
PI – 3.75 × 10^14^ cm^−2^	80	63	84	52
PI – 1 × 10^16^ cm^−2^	99	81	50	37

**Figure 9:**
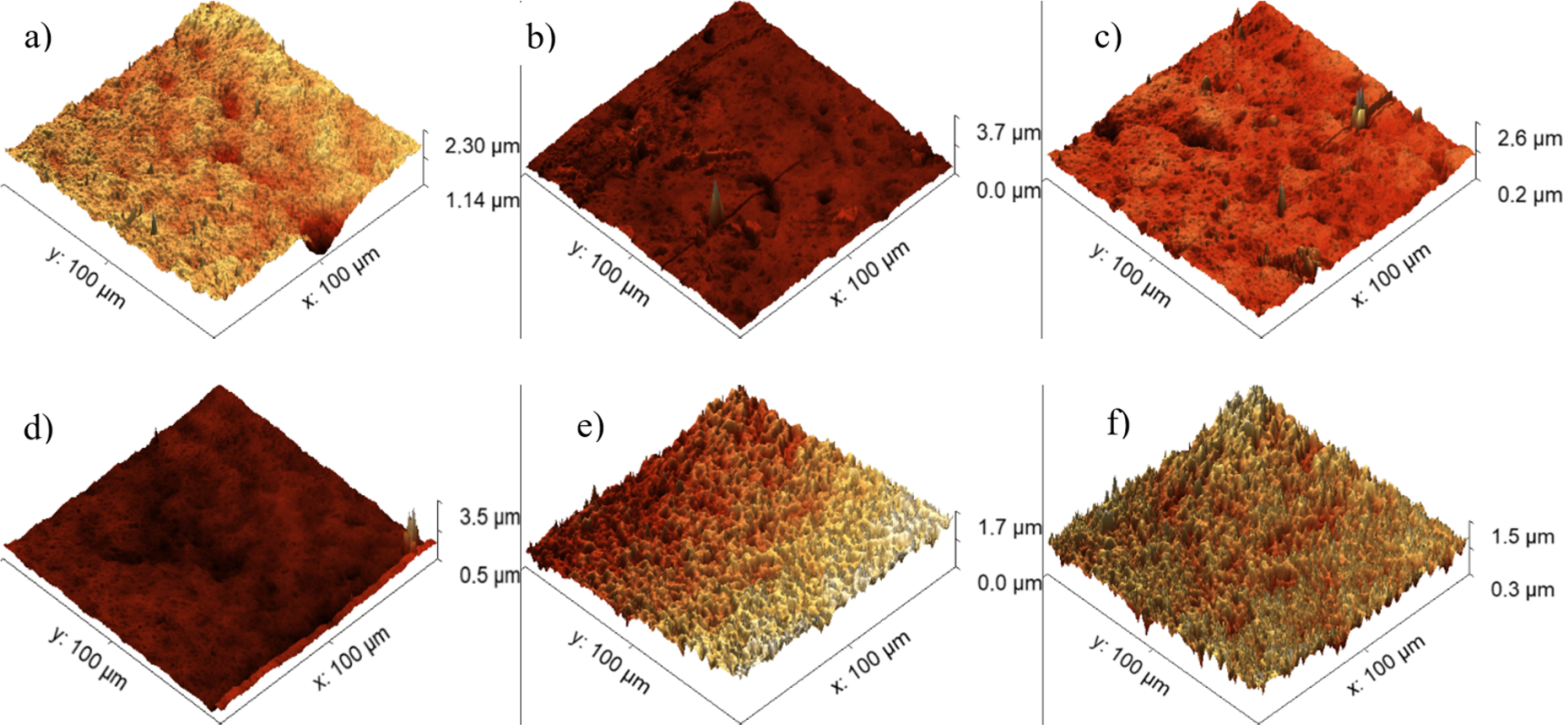
AFM images. (a) GO + Ag, 3.75 × 10^12^ cm^−2^, 20 keV; (b) GO + Ag, 3.75 × 10^14^ cm^−2^, 20 keV; (c) GO + Ag, 1 × 10^16^ cm^−2^, 20 keV; (d) GO + Ag, 3.75 × 10^12^ cm^−2^, 1.5 MeV; (e) GO + Ag, 3.75 × 10^14^ cm^−2^, 1.5 MeV; and (f) GO + Ag, 1 × 10^16^ cm^−2^, 1.5 MeV.

Figure 9 illustrates the alteration in the surface morphology of graphene oxide (GO) resulting from the implantation of 20 keV Ag ions (Figure 9) depending on the applied ion fluence. The minimal roughness was achieved when the GO sample was exposed to an ion fluence of 3.75 × 10^12^ cm^−2^, leading to a reduction in roughness to a minimum of 102 nm. Additionally, a discernible trend emerges indicating a substantial augmentation in roughness as the ion fluence increases. For instance, for an ion fluence of 3.75 × 10^14^ cm^−2^, the GO roughness increased 2.3-fold in comparison to the unmodified sample, and at the highest ion fluence of 1 × 10^16^ cm^−2^, the RMS parameter increased 3.8-fold compared to unmodified GO, reaching a value of 590 nm.

Silver ions at a medium energy of 1.5 MeV yielded different results. Implantation with a fluence of 3.75 × 10^12^ cm*^−^*^2^ led to a smoothing of the surface, which was reflected by a decrease in RMS to 102 nm and in *R*_a_ to 73 nm. At medium fluence of 3.75 × 10^14^ cm^−2^, the RMS increased slightly to 121 nm and the *R*_a_ to 96 nm, and these changes are not as pronounced as at lower energies. This suggests that the higher-energy ions penetrate deeper into the structure and affect the surface layer less. The highest fluence of 1 × 10^16^ cm^−2^ resulted in a decrease in RMS to 69 nm and of *R*_a_ to 60 nm, which may be due to surface compaction or reconstruction. Surface changes are less pronounced, probably due to deeper ion penetration. While higher fluences also distort the surface, the resulting densification or redistribution of defects may lead to a decrease in roughness at the highest fluence.

**Figure 10:**
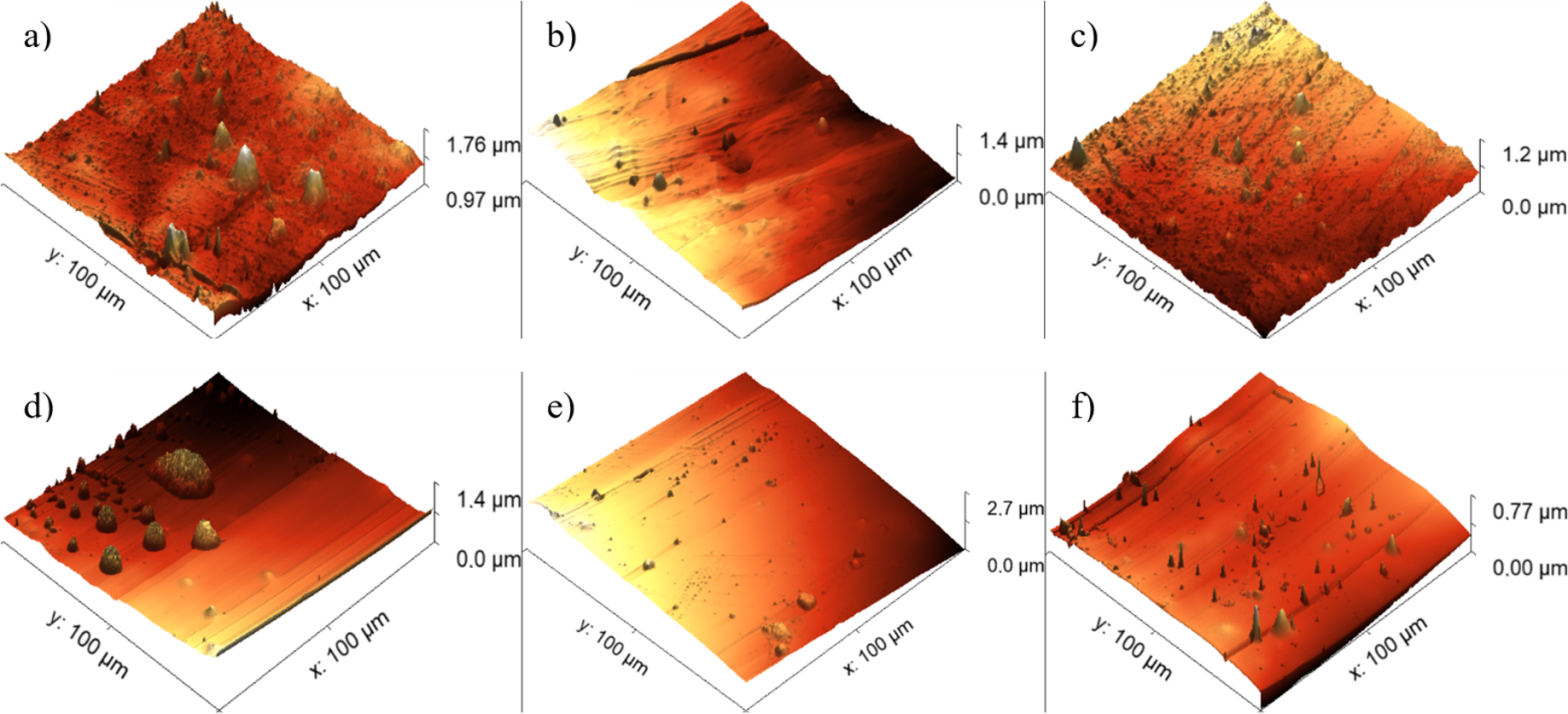
AFM images. (a) PI + Ag, 3.75×10^12^ cm^−2^, 20 keV; (b) PI + Ag, 3.75 × 10^14^ cm^−2^, 20 keV; (c) PI + Ag, 1 × 10^16^ cm^−2^, 20 keV; (d) PI + Ag, 3.75 × 10^12^ cm^−2^, 1.5 MeV; (e) PI + Ag, 3.75 × 10^14^ cm^−2^, 1.5 MeV; and (f) PI + Ag, 1 × 10^16^ cm^−2^, 1.5 MeV f).

The findings for PI suggest that the application of low-energy 20 keV Ag (Figure 10) ion implantation results in an overall decrease in surface roughness compared to the untreated material, regardless of the specific ion fluence. The minimum roughness was attained with an ion fluence of 3.75 × 10^12^ cm^−2^, measuring 64 nm. A comparable trend is evident when PI is implanted with 1.5 MeV Ag ions wherein a reduction in roughness is observable at any ion fluence relative to the reference condition. The lowest roughness in this instance was achieved with an ion fluence of 3.75 × 10^12^ cm^−2^.

One possible explanation for the change in surface roughness measured by AFM may be surface sputtering. When there is enough momentum transferred to the target, it can result in an atomic displacement from the solid, a process known as sputtering. There exists a critical energy level for sputtering to take place. Above this threshold, the sputter yield increases until it reaches a peak, after which it starts to decrease at even higher energies. This is because the ion penetrates the solid, preventing dislocated atoms from reaching the surface. The ion gradually loses energy to the atoms and becomes trapped [32].

### Photocatalytic properties

**Figure 11:**
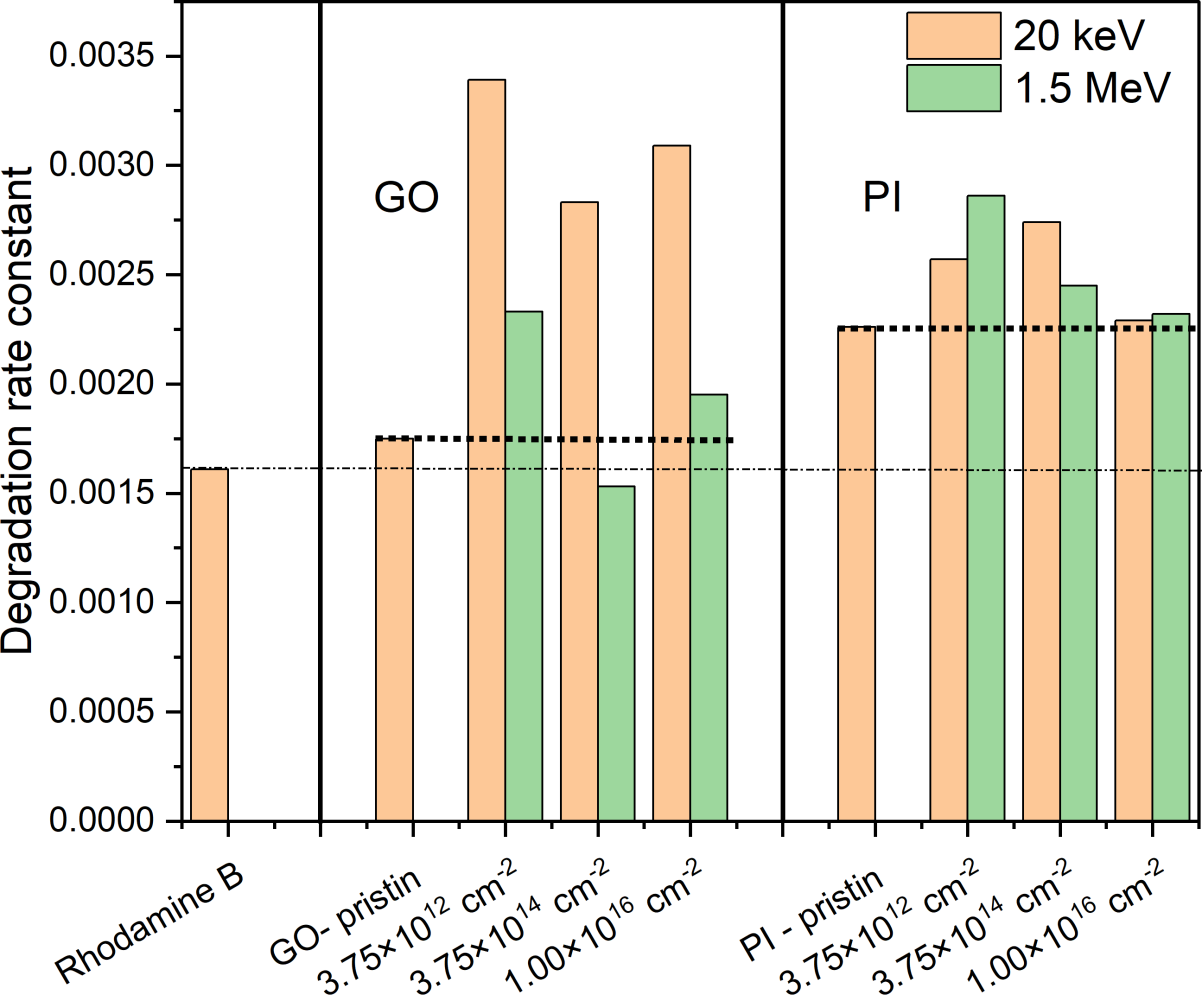
Evaluating the photocatalytic performance of both irradiated and non-irradiated GO and PI films through the degradation of rhodamine B under UV-irradiation.

The photocatalytic properties were tested in a dark chamber with UV light by degradation of the industrial dye rhodamine B in aqueous solution [33]. Results of photocatalytic testing of GO show different efficiencies depending on the energy and fluence of silver ions as you can see on the Figure 11.

Unmodified GO exhibits a lower rate constant for the decomposition of rhodamine B than the implanted samples. This suggests that the material itself without any intervention has limited ability to generate reactive oxygen species or facilitate charge transfer, which are both essential for efficient photocatalysis. There is a noticeable increase in the rate constant when low-energy Ag ions of 20 keV are implanted. This increase can be explained by the formation of defects in the GO surface layer, which increase the rate of electron excitation and facilitate subsequent charge transfer. At the same time, the implanted Ag ions can act as catalytic centers that improve the process of generating oxygen species. The low implantation energy results in the ions remaining relatively close to the surface, where they can most influence the chemical reactivity of the material.

A different trend is observed when GO is implanted with silver ions with a mean energy of 1.5 MeV. At a fluence of 3.75 × 10^12^ cm^−2^, there is a slight increase in photocatalytic activity, but the value is significantly lower than at the same fluence with an energy of 20 keV. A similar decrease in efficiency was observed at higher fluences. It is evident that roughness can affect the photocatalytic properties of GO as seen from the AFM readings. Implantation of 20 keV Ag ions showed a significant increase in roughness compared to implantation with 1.5 MeV Ag ions.

The degradation rate constant was also increased in the case of PI implantation (Figure 11), but the increase is not as significant as in the case of GO. The highest increase in the degradation rate constant is evident for PI implanted with 1.5 MeV Ag ions with a fluence of 3.75 × 10^12^ cm^−2^. The formation of defects in the main polymer chain can lead to the binding of Ag ions to the polymer chain structure. Ag ions can bind mainly as oxides, which improves the semiconducting properties of the polymer matrix and reduces the bandgap width. Following the PI implantation with the lowest fluence, the Ag ions have the ability to bond with the newly created free bonds (C–C), resulting in the formation of oxides [21]. This process can lead to a reduction in the bandgap and consequently enhance the photocatalytic power [34].

Another process that may influence the photocatalytic degradation of rhodamine B is the presence of free radicals. Free radicals are generated during ion implantation in which the implanted ion significantly ionizes its surroundings, resulting in the formation of free radicals. Free radicals are among the highly reactive particles that may be involved in the degradation of rhodamine B [11].

AFM analysis revealed that Ag^+^ ion implantation leads to pronounced changes in surface morphology, with the degree of roughness depending on both ion fluence and implantation energy. A comparison of these morphological characteristics with the photocatalytic results shows that the highest efficiency is achieved using samples with moderate nanoscale roughness, that is, GO implanted with 20 keV at a fluence of 3.75 × 10^12^ cm^−2^ (RMS = 102 nm, *k* ≈ 0.0033 min^−1^) and PI implanted with 20 keV at the same fluence (RMS = 64 nm). Such surfaces likely provide an optimal balance between the density of active sites and efficient charge carrier transport, thereby reducing recombination. In contrast, excessive roughening, as observed for GO at 1 × 10^16^ cm^−2^ (RMS = 590 nm), did not result in further improvement and may be associated with the formation of defects that promote recombination.

A similar trend was observed for implantation at 1.5 MeV, although the differences in roughness between samples were less pronounced. The best performance was still recorded for samples with the lowest fluence (GO and PI, 3.75 × 10^12^ cm^−2^), suggesting that, at greater implantation depths, subsurface effects and Ag-induced electronic modifications play a more important role than surface topography alone. These results indicate that the key to maximizing photocatalytic efficiency is not to maximize roughness, but to achieve a balanced nanoscale surface structure that complements the plasmonic and electronic effects of silver, enabling a high density of accessible active sites while maintaining efficient charge separation.

### Electric and sensory properties

**Figure 12:**
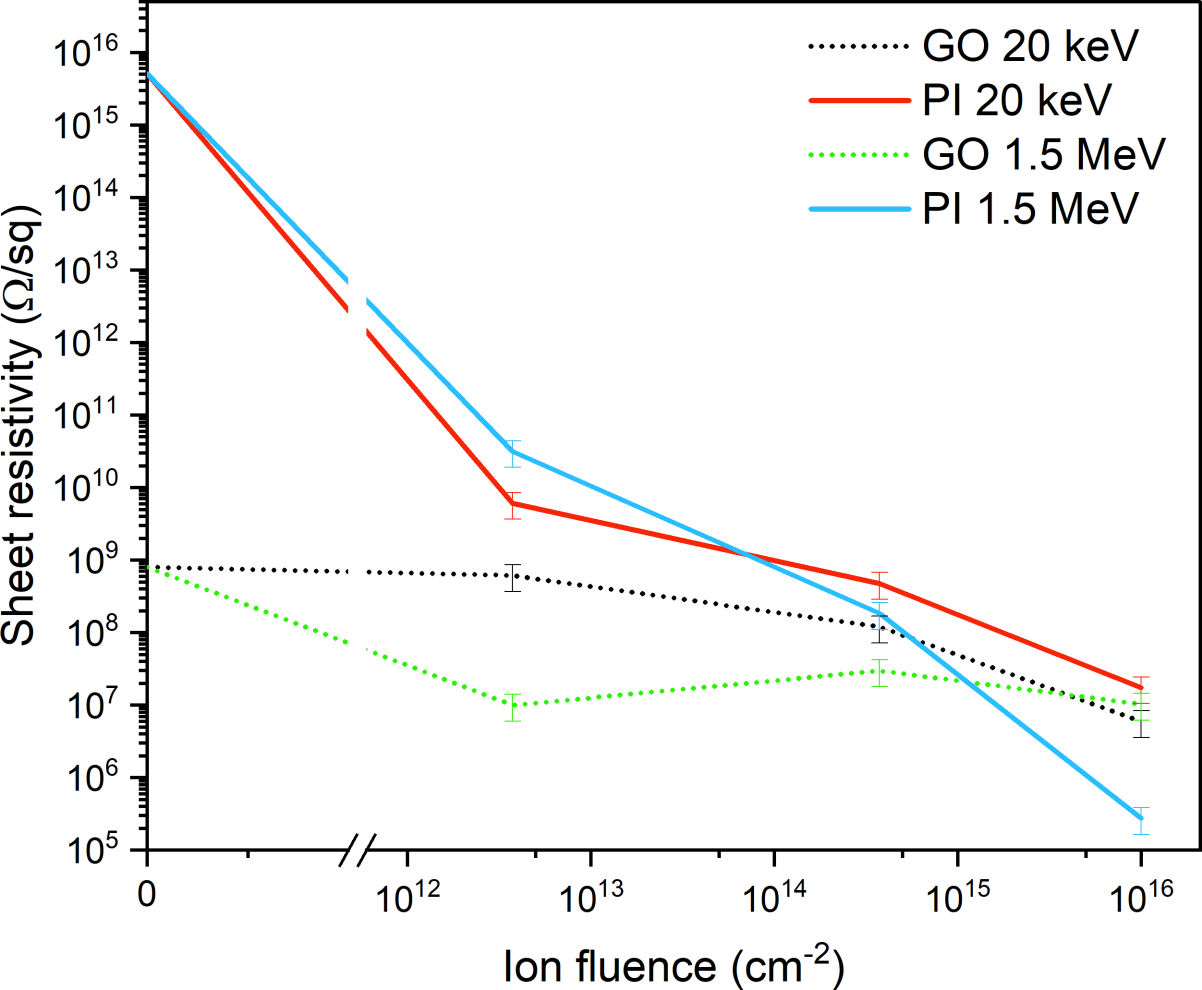
The sheet resistivity of GO and PI after implantation using 20 keV and 1.5 MeV Ag ions with ion fluences of 3.75 × 10^12^ cm^−2^, 3.75 × 10^14^ cm^−2^ and 1 × 10^16^ cm^−2^.

The results of electrical property measurements are illustrated in Figure 12, showing the correlation between sheet resistivity and applied ion fluence. The data indicates that unmodified GO exhibits a sheet resistivity of 1 × 10^9^ Ω·sq^−1^, which decreases after ion implantation with increasing ion fluence. GO implanted with both energies of Ag ions has the minimal sheet resistivity (1 × 10^7^ Ω·sq^−1^) after implantation using the ion fluence of 1 × 10^16^ cm^−2^.

GO implanted with 20 keV Ag ions shows a slight decrease in surface resistivity with increasing ion fluence. This can be explained by the intrinsic structure of GO, which, already in the unaltered state, contains certain conductive regions and functional groups that affect its electronic properties. The lowest values of surface resistivity were achieved at an ionic fluence of 1 × 10^16^ cm^−2^, when the surface resistivity decreases to 1 × 10^8^ Ω·sq^−1^. A similar trend is seen in the case of GO implantation with 1.5 MeV Ag ions. GO contains many oxygen functional groups (epoxide, hydroxy, and carbonyl) which disrupt the continuous sp^2^-hybridized network of carbon atoms and cause high electrical resistivity. Ionic implantation with high fluence gradually removes these functional groups, which is confirmed by XPS analysis showing a decrease in the C=O and C–O content.

In the case of a PI implanted with silver ions with an energy of 20 keV, a systematic decrease in surface resistivity can be observed as a function of increasing ionic fluence. At low fluence (3.75 × 10^12^ cm^−2^), limited modification of the polyimide surface layer occurs, the main mechanism being defect formation and partial cleavage of the polymer chains. However, the resulting graphitic regions are not yet sufficiently interconnected and, therefore, the surface resistance remains relatively high. As the fluence increases to 3.75 × 10^14^ cm^−2^, the graphitization of the material becomes more intense, as suggested by the RBS results, leading to a more pronounced decrease in resistance by several orders of magnitude. A more continuous network of conductive pathways is formed, enabling more efficient charge transport through the material. At the highest implanted fluence of 10^16^ cm^−2^, the polyimide is already extensively transformed, causing a decrease in resistance to values as low as 1 × 10^8^ Ω·sq^−1^, which is also due to the significant sp^2^ hybridization.

In the case of implantation of silver ions with a medium energy of 1.5 MeV, the decrease in surface resistance is much more pronounced, which corresponds to the implantation depth. While at 20 keV, the modification occurs mainly in the surface layer, implantation at 1.5 MeV allows the ions to penetrate deep into the polyimide structure, as confirmed by RBS and SRIM simulations. Already at low fluence (3.75 × 10^12^ cm^−2^), a decrease in surface resistivity is seen, suggesting that deep energy deposition induces extensive fragmentation of the polymer skeleton, in particular cleavage of C–N, C–O, and C=C bonds and more efficient formation of conducting phases. As the fluence increases to 3.75 × 10^14^ cm^−2^, the resistivity further decreases sharply as the density of graphitic structures increases and an interconnected network of carbon-carbon regions is formed throughout the material volume, as suggested by the XPS results. At the highest fluence of 1 × 10^16^ cm^−2^, the surface resistivity reaches minimum values because the polyimide is almost completely transformed into a conductive structure with a high proportion of graphitized regions and metallic silver nanoclusters. Compared to low-energy implantation, the conductivity effect here is much stronger due to the bulk effect, leading to a decrease in resistance of up to eleven orders of magnitude.

**Figure 13:**
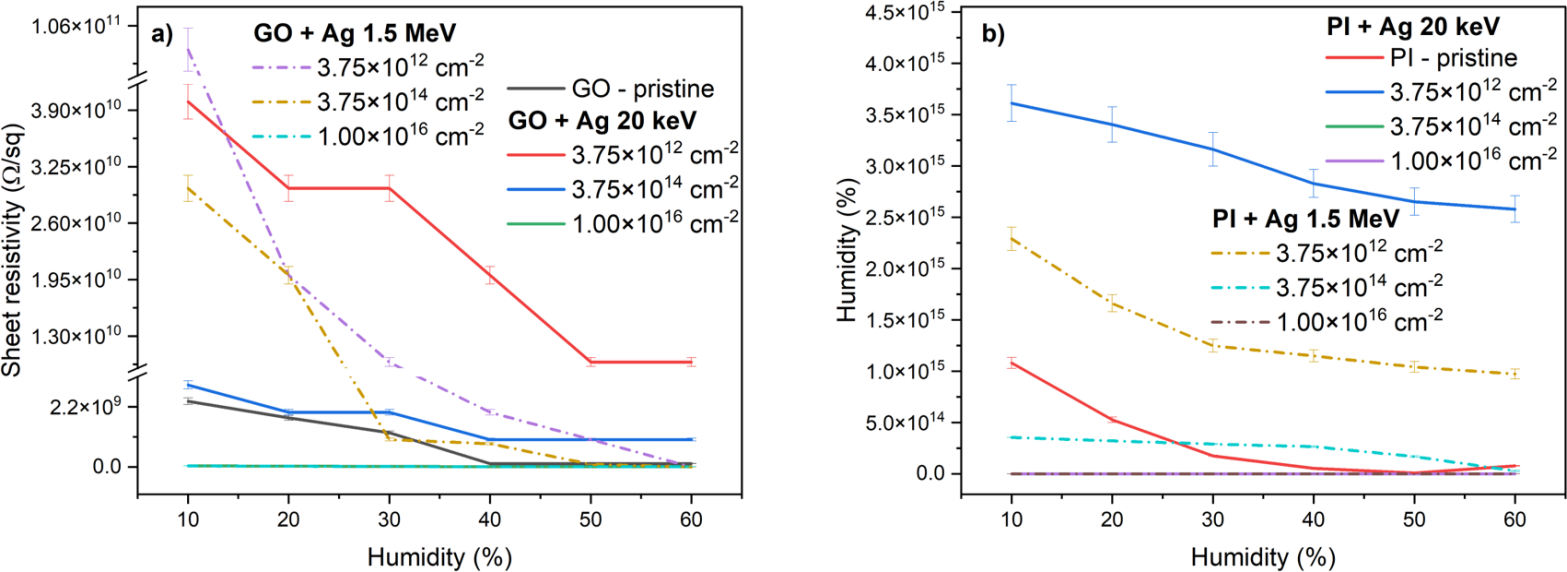
Dependence of the sheet resistivity of pristine and implanted (a) GO and (b) PI after irradiation different energies and ion fluences on air humidity in an atmospheric chamber.

The sensory properties of the modified and unmodified samples were determined by measuring the changes in sheet resistivity as a function of the relative humidity in an atmospheric chamber. From the results in Figure 13a, where the dependence of the sheet resistivity is shown, it is evident that the unmodified GO changes the sheet resistivity value only at 40% relative humidity.

The sensitivity of graphene oxide to atmospheric humidity increases after implantation of Ag ions with both energies used, 20 keV and 1.5 MeV. The sample sensitivity is demonstrated by a decrease in surface resistivity with increasing air humidity. The best results were obtained for GO implanted with Ag ions at an energy of 1.5 MeV and an ion fluence of 3.75 × 10^12^ cm^−2^. For this sample, the accompanying graph (Figure 13a) shows a gradual linear decrease in the sheet resistivity over the entire range of relative humidity measurements. The sheet resistivity of this sample at 10% relative humidity was 1 × 10^11^ Ω·sq^−1^, while after increasing the relative humidity to the 60%, the sheet resistivity decreased significantly to 2 × 10^8^ Ω·sq^−1^. Different results were observed for the PI foils implanted with Ag ions (Figure 13b), where only the sample implanted with the lowest fluence of ions at an energy of 1.5 MeV exhibits sensory sensitivity. The results for PI show that the optimal response to relative humidity change was only achieved for samples implanted at 20 keV with fluence of 3.75 × 10^12^ cm^−2^ and for 1.5 MeV Ag ions with fluence 3.75 × 10^12^ cm^−2^. These samples show a gradual decrease in surface resistivity with increasing humidity, suggesting that ion implantation has modified the material in a way that promotes interaction with water molecules and allows for a dynamic change in electrical properties. Conversely, samples with higher fluence no longer show a significant dependence of resistance on humidity content. This suggests that, at high fluences, excessive carbonization of the material or the formation of a continuous conductive pathway occurs, which suppresses the effects of water adsorption on charge transport.

## Conclusion

The aim of this work was to improve the sensing, electrical, and photocatalytic properties of GO and PI implanted with Ag ions in the ion fluence range from 3.75 × 10^12^ cm^−2^ to 1 × 10^16^ cm^−2^. We used low energies (20 keV) to modify the subsurface layers to a depth of 30 nm. Furthermore, we used energies of 1.5 MeV to ensure modification of the subsurface layers to a depth of 800 nm. The results show that increasing ion fluence leads to an increase in the atomic concentration of carbon and a decrease in non-carbon elements such as oxygen and hydrogen in most cases (GO and PI). The most pronounced changes were achieved at an energy of 1.5 MeV and an ion fluence of 1 × 10^16^ cm^−2^. A similar trend was confirmed by XPS. We also observed changes in roughness as a result of ion irradiation. It was shown that the implantation of Ag ions into PI (20 keV, 1.5 MeV) leads to a significant increase in its roughness. The most pronounced decrease in roughness was observed for GO implanted at 1.5 MeV with an ion fluence of 1 × 10^16^ cm^−2^, where the roughness decreased by almost 85 nm.

The experimental results demonstrate that the optimal ion fluence for enhancing the humidity sensing and photocatalytic properties of GO and PI films is 3.75 × 10^12^ cm^−2^ at an energy 20 keV. This fluence level induces the formation of conjugated bonds in the surface layers of GO and PI, as evidenced by RBS and XPS analysis. The electrical conductivity of GO films remains relatively stable with increasing ion fluence, likely due to the inherently low resistivity of pristine GO. The most significant reduction in surface resistivity, which reaches ten orders of magnitude compared to pristine PI, was observed for PI modified at 1.5 MeV with an ion fluence of 1 × 10^16^ cm^−2^. This significant decrease in resistivity can be attributed to the formation of conductive pathways within the PI matrix, effectively enhancing its electrical conductivity.

Additionally, the study suggests that the photocatalytic properties of GO and PI films are most effective for the lowest ion fluence of Ag ions (3.75 × 10^12^ cm^−2^) at an energy of 20 keV, indicating that a delicate balance between the formation of conjugated bonds and the preservation of GO inherent properties is crucial for maximizing both sensing and photocatalytic performance. These findings highlight the potential of Ag ion implantation to significantly enhance the functionalities of GO and PI films for various applications, including environmental monitoring and photocatalysis.

## Experimental

Hummers’ method was employed to synthesize graphene oxide (GO), following the procedure outlined in [16]. GO film was prepared through filtration using a polycarbonate membrane (Nucleopore 45 μm, 90 mm in diameter). The density of the resulting film was measured as 1.36 g·cm^−2^. An additional material incorporated in the study was polyimide (PI) with a density 1.42 g·cm^−3^ and a surface resistance above 10^14^ Ω·sq^−1^, which was supplied by Goodfellow [9–10]. Both substrates, GO and PI, were exposed to low-energy ion implantation with 20 keV Ag^+^ ions using a 40 keV ion implanter at Helmholtz Zentrum Dresden-Rossendorf, Germany. Midle-energy ion implantation with 1.5 MeV Ag^+^ ions was performed using an ion accelerator (Tandetron 4130 MC) at Nuclear Physics Institute of the CAS, Rez, Czechia. The ion implantation was performed at room temperature. GO and PI were implanted with Ag^+^ ions at fluences of 3.75 × 10^12^ cm^−2^, 3.75 × 10^14^ cm^−2^, and 1.0 × 10^16^ cm^−2^. The ion current during the implantation was kept at approximately 20–45 nA·cm^−2^.

The elemental composition of implanted and pristine samples of GO and PI was analyzed using RBS and ERDA methods. The elemental concentrations of C, O, and Ag were analyzed from the RBS spectra collected using 2.0 and 3.07 MeV He^+^ ions. The RBS spectra for Ag depth profiling were obtained using 2.0 MeV protons. The RBS spectra were recorded using an Ultra-Ortec PIPS detector. The projectile He^+^ ions were backscattered at a laboratory scattering angle of 170° in a Cornell geometry. The H concentration was analyzed by the ERDA method using He^+^ ions with an energy 2.5 MeV. The ERDA primary beam incoming angle was 75°, and the scattering angle was 30°. The recoiled particles were recorded by a Canberra PIPS detector covered by 12 μm thick Mylar foil. To avoid sample damage and modification during RBS analysis, the measurements were performed at multiple sample locations. The elemental concentration was analyzed using the SIMNRA code [19].

The electronic structure and chemical composition of the surface layers were analyzed by XPS, with an Omicron Nanotechnology ESCAProbeP spectrometer. The X-ray monochromatic source at 1486.7 eV was used and XPS spectra were evaluated using CasaXPS software.

The structural analysis of irradiated GO and PI was investigated through Raman and attenuated total reflectance Fourier-transform infrared (ATR-FTIR) spectroscopies. Raman spectroscopy was conducted using a Renishaw inVia Raman microscope (England) equipped with a Nd:YAG laser (532 nm, 50 mW) and a 50× magnification objective. For ATR-FTIR measurements, a NICOLET iS50R FTIR spectrometer (Thermo Scientific, USA) was employed. The measurements, spanning the range of 4000–400 cm^−1^ at a resolution of 4 cm^−1^, utilized a diamond ATR crystal and a DTGS detector for precise analysis.

The surface morphology and roughness of the non-implanted and implanted samples were measured using AFM (Dimension ICON, Bruker) in quantitative nanoscale mechanical (QNM) mode under ambient conditions. A SCANASYST-AIR tip made of nitride with an elastic constant of 0.4 N·m^−1^ was utilized for the measurements. The acquired AFM data were processed using NanoScope Analysis software.

The photocatalytic properties were evaluated by rhodamine B degradation under UV light within a dark chamber. In this setup, the prepared samples were submerged in a rhodamine B aqueous solution at a concentration of 5 mg·L^−1^ and exposed to UV light (254 nm, 40 W) for 180 min. Subsequently, the rhodamine B concentration and disintegration were examined using absorption spectra via a UVISEL ellipsometer (75 W Xe lamp, Horiba, France) in the 450–650 nm wavelength range with a 1 nm step. The ellipsometer beam spot size was 1 nm.

During the absorption measurements, the degradation constant was determined by assessing the ratio of the rhodamine B concentration in the analyte to that of the initial rhodamine B solution over time. The degradation constant associated with photocatalytic degradation was derived using the first-order kinetic equation:


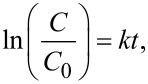


where *C* represents the final concentration at time *t, C*_0_ is the initial concentration of rhodamine B, and *k* is the apparent rate constant of degradation [11]. The relation ln(*A*/*A*_0_) was used to represent the degradation rate kinetics, where *A* is the measured optical absorbance integrated over the 450–650 nm wavelength range, and *A*_0_ is the nominal optical activity of the pure rhodamine B solution [20].

Humidity-sensing capabilities were investigated by measuring the electric properties of modified GO and PI surfaces in a climatic chamber. The chamber’s atmosphere relative humidity (controlled in the range of 5–60% RH) was maintained by blending moist and dry air, monitored using a BME 280 pressure/humidity/temperature sensor.

The electrical properties of the implanted and pristine samples were studied by the standard two-point method, where the sheet resistance was measured using a Keithley 6317B electrometer. To quantify the sheet resistivity, two gold contacts, each with a thickness of 50 nm and a length of 10 mm, were deposited onto the surfaces of GO and PI thin films. The placement of these contacts adhered to a prescribed contact distance of 1 mm, ensuring a consistent and controlled measurement configuration.

## Data Availability

Data generated and analyzed during this study is openly available in Zenodo at https://doi.org/10.5281/zenodo.15631026
